# Solvent-controlled regioselective protection of allyl-4,6-benzylidene glucopyranosides

**DOI:** 10.1186/1860-5397-3-26

**Published:** 2007-09-26

**Authors:** Kerry Ann Ness, Marie E Migaud

**Affiliations:** 1 School of Chemistry and Chemical Engineering, Stranmillis Road, Queens University, Belfast, BT9 5AG, Northern Ireland, UK

## Abstract

We wish to report a simple synthetic procedure, which permits the regiospecific mono-acylation, alkylation and silylation at the 2-position of allyl 4,6-*O*-benzylidene α-D-glucopyranoside in high yields and which does not require the use of catalysts.

## Background

Numerous syntheses of oligosaccharides incorporating glucose moieties have been reported. In most cases, a limiting synthetic factor is the number of functional group manipulations required to access suitable synthetic precursors. For hexopyranoses, acylation of cis-diols can be achieved with high regioselectivity either by means of metal activators such as tin [1–3], silver [4], boron [5] or copper [6] or by exploiting the relative reactivity of hydroxyl groups [7–8]. However, metal-promoted alkylation and base-catalysed acylation of diols have proven to be highly undependable in the case of glucose and other cyclic trans-diols, where both hydroxyl groups are equatorial. For instance, reports of identical procedures describing the tin-catalysed benzylation of methyl 4,6-*O*-benzylidene glucopyranoside claim isolated yields ranging from the 37% and below [9] to 75% and above [10]. Others reported multi-step procedures to achieve introduction of a suitable protecting group at the 2-position of the 4,6-*O*-benzylidene 1-*O*-alkyl protected glucose [11] or used enzymes to achieve selectivity [12].

## Results and discussion

**Scheme 1:**
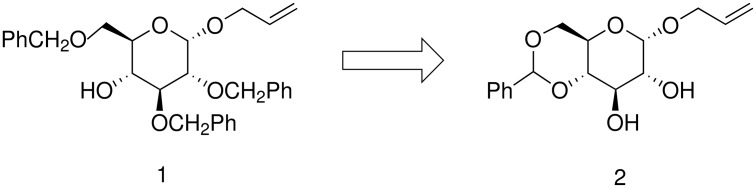
Target partially protected sugar **1**.

**Scheme 2:**
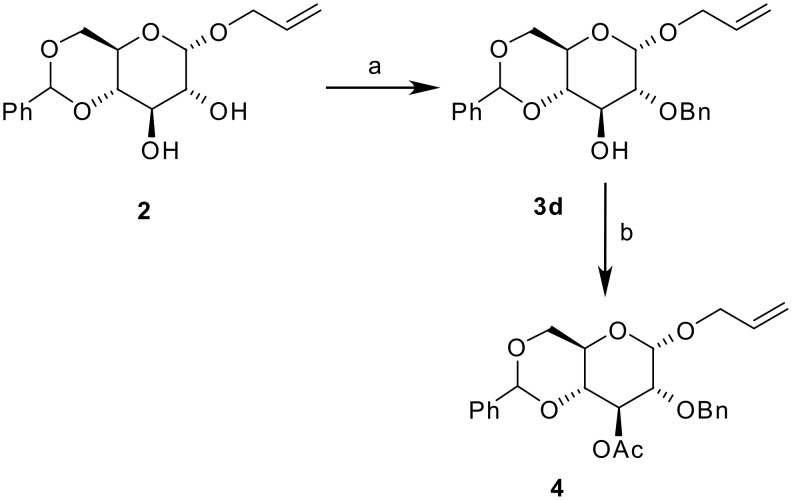
Selective C-2 benzylation and confirmation of the regiochemistry through acetylation of the C-3 hydroxyl. a. BnBr, NaH, Bu_4_NI, THF, 80°C; b. Ac_2_O, DMAP, Py, MW, 5 mins, 80 W

While preparing the partially protected glucose **1** from α-allyl-4,6-benzylidene glucoside **2** (Scheme 1), we observed that mono-benzylation could be achieved, if instead of DMF and the usual reagents' combination (i.e. NaH, BnBr, Bu_4_NI), THF was to be used as reaction solvent (Scheme 1). Osborn had reported the regioselective mono-acylation/alkylation of the C-3 hydroxyl of 4,6-*O*-benzylidene-β-D-glycopyranosides using NaH/CuCl_2_ in THF [6]. Distinctively, we observed the regioselective benzylation at the C-2 position of the 1-*O*-allyl-α-glucoside **2** (Scheme 2). This assignment was in agreement with previously published NMR data [11,13] and confirmed by acetylation of the mono-protected material **3d**, to give compound **4**, which resulted in an H-3 NMR shift from 4.15 ppm to 5.51 ppm.

Introduction of other protecting groups were then considered. Alkylation, acylation and silylation using halogenated reagents offered mono-protection when reactions were carried out in THF and regio-selectivity was achieved when large protecting groups were employed (Table 1) (see Supporting Information File 1 for full experimental data). In most cases, the expected products could not be obtained when DMF was used as solvent.

**Table 1:** Reaction of **2** with alkylating, acylating and silylating reagents and products distribution.

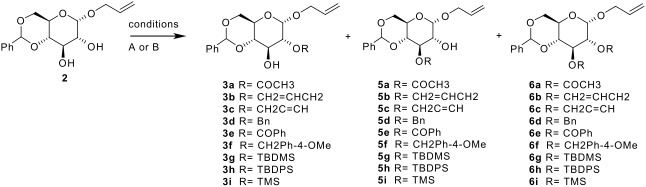							

Product type	Reagent	Conditions^a^	Crude yield %	**2** isolated yield %	**3** isolated yield %	**5** isolated yield %	**6** isolated yield %

a	CH_3_COCl	THF	98	22	36	36	-
a	CH_3_COCl	DMF	99	20	-	-	75
b	CH_2_ = CHCH_2_Br	THF	95	25	31	31	-
b	CH_2_ = CHCH_2_Br	DMF	97	26	-	-	64
c	HC ≡ CCH_2_Br	THF	89	21	43	32	-
c	HC ≡ CCH_2_Br	DMF	90	26	-	-	68
d	BnBr	THF	93	23	68	-	-
d	BnBr	DMF	95	22	-	-	76
e	PhCOBr	THF	92	32	57	-	-
e	PhCOBr	DMF	97	28	-	-	62
f	PMBCl	THF	94	30	56	-	
f	PMBCl	DMF	85	34	-	-	63
g	TBDMSCl	THF	88	23	52	-	-
g	TBDMSCl	DMF	92	92	-	-	-
h	TBDPSCl	THF	97	48	45	-	-
h	TBDPSCl	DMF	96	96	-	-	-
i	TMSCl	THF	96	44	50	-	-
i	TMSCl	DMF	97	97	-	-	-

^a^ A: THF; 70°C, 16 hours, 3.5 eq RCl, 4.5 eq NaH, Bu_4_NI, 0.024 M; B: DMF; 70°C, 16 hours, 3.5 eq RCl, 4.5 eq NaH, 0.024 M.

Two conclusions could be drawn. Firstly, mono-alkylation of allyl 4,6-*O*-benzylidene α-D-glucopyranoside could be achieved in THF under concentrated solution conditions, even in the presence of an excess of base and alkylating reagent. Secondly, regioselectivity was achieved if the alkylating reagent was bulky (Table 1). When both allyl bromide and acetyl chloride were used for the reaction in THF a mixture of the C-2 and C-3 mono-protected products were formed. The smaller protecting groups do not encounter the same steric hindrance as the larger groups due to the benzylidene ring. Yet no bis-protected product is formed with these reagents under these conditions, suggesting that once one hydroxyl has reacted to give the mono-protected product, the other hydroxyl must be deactivated so that no further reaction occurs.

In order to rationalise such regioselectivity, alkylation and silylation reactions of other 4,6-benzylidene protected glycosides were carried out (Table 2, Table 3, Table 4). The reaction carried out with DMF as solvent gave the bis-protected galactosides when PMBCl was used and no reaction when the silylating reagents were used. In THF, alkylation occurred with similar outcomes to that observed in DMF. However, THF offered means to access the monosilylated galactosides **8** and **9**, even though in very modest yields. This change in reactivity in THF can be linked to the change in the hydrogen-binding pattern of the sugar and the resulting acidity of the alcohol groups. The steric and electronic effect of the anomeric substituents was then assessed by examining the β-allyl glucoside anomer (Table 3). The allyl-4,6-*O-*benzylidene-β-D-glucopyranoside, **11** was synthesised from 2,3,4,6-tetra-*O*-acetyl-α-D-glucopyranosyl bromide using mercury bromide, mercury oxide and allyl alcohol. Only dialkylated material **14** was obtained under both sets of alkylation conditions. To examine the impact of the nature of the glycon, benzylation of methyl and benzyl 4,6-benzylidene-α-D-glucosides, **15** and **16** respectively, was also carried out (Table 4). The reaction also yielded the 2,3-di-benzylated α-D-glucosides. These results differ greatly from these obtained for the α-allyl glucoside **2** where only mono-benzylation was achieved. This change in reactivity between the glucose derivatives shows the dramatic effect that the aglycon group has on the alcohols' reactivity under basic conditions. It appears that the regioselectivity observed in THF for the glucoside **2** was directed by three factors, the nature of the halogenated reagent (steric effect), the hydrogen bond network created by the alpha configuration and finally while unexpectedly the presence of an allyl ether at the C1 position of the glucoside.

**Table 2:** Reaction of galactoside **7** with alkylating and silylating reagents and products distribution.

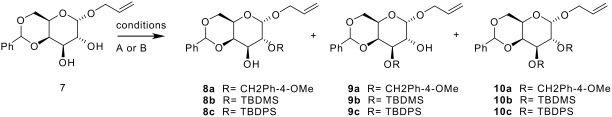							

Product type	Reagent	Conditions^a^	Crude yield %	**7** isolated yield %	**8** isolated yield %	**9** isolated yield %	**10** isolated yield %

a	PMBCl	THF	96	30	-	-	60
a	PMBCl	DMF	95	20	-	-	75
b	TBDMSCl	THF	92	15	38	35	-
b	TBDMSCl	DMF	93	65	-	-	-
c	TBDPSCl	THF	90	48	20	20	-
c	TBDPSCl	DMF	85	68	-	-	-

^a^ A: THF; 70°C, 16 hours, 3.5 eq RCl, 4.5 eq NaH, Bu_4_NI, 0.024 M; B: DMF; 70°C, 16 hours, 3.5 eq RCl, 4.5 eq NaH, 0.024 M.

**Table 3:** Reaction of glucoside **11** with benzyl halide and products distribution.

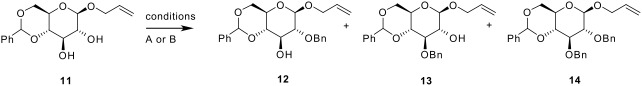						

Sugar	Conditions^a^	Crude yield- %	**11** isolated yield %	**12** isolated yield %	**13** isolated yield %	**14** isolated yield %

**11**	THF	92	23	-	-	64
**11**	DMF	95	20	-	-	68

^a^ A: THF; 70°C, 16 hours, 3.5 eq BnBr, 4.5 eq NaH, Bu_4_NI, 0.024 M; B: DMF; 70°C, 16 hours, 3.5 eq BnBr, 4.5 eq NaH, 0.024 M.

**Table 4:** Reaction of glucoside **15** and **16** with benzyl halide and products distribution.

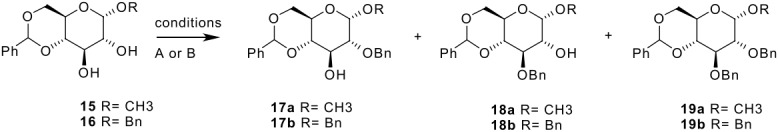							

Product type	Sugar	Conditions^a^	Crude yield- %	**15** or **16** isolated yield %	**17** isolated yield %	**18** isolated yield %	**19** isolated yield %

a	**15**	THF	87	26	-	-	60
a	**15**	DMF	90	21	-	-	70
b	**16**	THF	93	21	-	-	68
b	**16**	DMF	95	19	-	-	75

^a^ A: THF; 70°C, 16 hours, 3.5 eq BnBr, 4.5 eq NaH, Bu_4_NI, 0.024 M; B: DMF; 70°C, 16 hours, 3.5 eq BnBr, 4.5 eq NaH, 0.024 M.

It can be postulated that in THF, regioselectivity depends on the relative acidity of the secondary hydroxyl groups and the nucleophilicity of the resulting alkoxide. The acidity is modulated by intramolecular H-bonds while steric effects control the nucleophilicity. Vasella [7] and Moitessier [8], both reported on the strength and the effects of cis- and trans-intramolecular H-bonds within protected glucose derivatives. The H-bond between the C2-hydroxyl and the α-C1-allyloxy in the partially protected glucoside **2** renders the C2-hydroxyl group the more acidic of the two free hydroxyls. In galactoside **7**, the C3-hydroxyl group is capable of forming strong hydrogen bonding interaction with the cis C4-vicinal oxygen. Here, both C2- and C3-hydroxyls have similar chemical reactivity as both are involved in cis-H-bonds with vicinal oxygens. Mono-alkylation, acylation and silylation of allylated glucoside **2** and galactoside **6** in THF could be related to the poor solvation of the conjugated alkoxides and decreased reactivity to that compared in a more polar solvent such as DMF. However, the multiple substitutions obtained both in DMF and THF for the methyl and benzyl glucosides **15** and **16** would indicate that the selectivity obtained in glucoside **2** and galactoside **7** relied on the nature of the protecting group at the C-1 position, i.e. the allyl group.

In summary, we have stumbled on a very simple, yet very versatile and high yielding method to specifically protect the C2-hydroxyl group of α-allyl-glucoside, which does not require any form of activators. It can be anticipated that this method will share itself to the introduction of moieties other than protecting groups, such as hindered alkyl and silyl halides or acylchlorides of carbohydrate derivatives.

## Supporting Information

File 1 experimental section. The data provided describes the procedures employed to complete the synthetic work.

## References

[1] Peri F, Cipolla L, Nicotra F (2000). Tetrahedron Lett.

[2] Tsuda Y (1997). J Synth Org Chem, Jpn.

[3] Grindley T B (1998). Adv Carbohydr Chem Biochem.

[4] Wang H S, She J, Zhang L H, Ye X S (2004). J Org Chem.

[5] Oshima K, Kitazono E, Aoyama Y (1997). Tetrahedron Lett.

[6] Osborn H M I, Brome V A, Harwood L M, Suthers W G (2001). Carbohydr Res.

[7] Hu G X, Vasella A (2002). Helv Chim Acta.

[8] Moitessier N, Chapleur Y (2003). Tetrahedron Lett.

[9] Chen J, Dorman G, Prestwich G D (1996). J Org Chem.

[10] Liu D S, Chen R, Hong L W, Sofia M J (1998). Tetrahedron Lett.

[11] Dong L, Roosenberg J M, Miller M J (2002). J Am Chem Soc.

[12] Nahmany M, Melman A (2004). Org Biomol Chem.

[13] Zhang S-Q, Li Z-J, Wang A-B, Cai M-S, Feng Rui (1998). Carbohydr Res.

