# Supplementary material for: Solvent-controlled regioselective protection of allyl-4,6-benzylidene glucopyranosides
Source: Beilstein J Org Chem. 2007 Sep 26;3:26. doi: 10.1186/1860-5397-3-26 (PMC2048501; doi:10.1186/1860-5397-3-26)
Supplement: File 1 — experimental section. The data provided describes the procedures employed to complete the synthetic work. [file Beilstein_J_Org_Chem-03-26-s001.doc]

**Experimental.**

**General Experimental**

The 1H, and 13C NMR spectra were recorded in CDCl3 or D2O on a Brüker AC 300MHz spectrometer. HMQC and 1H-1H COSY experiments were performed on a Brüker AMX 500MHz spectrometer. The chemical shifts are recorded in δ p.p.m. and *J* values recorded in Hz. TMS (0ppm, 1H NMR) and CHCl3 (77ppm, 13C) were used as internal standards. Optical rotation were determined with a Perkin Elmer 341 polarimeter. Liquid secondary ion mass spectra (LSIMS) were recorded in a 3-nitrobenzyl matrix with an effective accelerating voltage of 30kV on a VG Autospec (Micromass, UK) instrument. Electrospray mass spectra (ES-MS) was performed on a VG Quattro Triple Quadrupole Mass Spectrometer with a flow rate of 20μl/min and a capillary voltage of 3.5kV. Reactions were carried out under dry argon atmosphere.

**Allyl-α-***D***-glucopyranoside** and **allyl-β-***D***-glucopyranoside[1]**

*D*-Glucose (0.10g, 0.55mmol) was partially dissolved in allyl alcohol (3ml, 44.0mmol). To this suspension *para*-toluenesulphonic acid (0.01g, 0.06mmol) was then added and the resulting suspension stirred for 16 hours at 90oC. Diethyl ether (5ml) was added and the mixture was left to sit overnight. This was then evaporated off and the crude material purified by column chromatography and the resulting product azeotroped with hexane to remove allyl alcohol yielding 0.06g (52%) of pure material a colourless syrup.

1H NMR: (500MHz, MeOD)-δ5.61-5.60(m, 1H, 2xAll-2-H), 4.97(d, 1H, 2xAll-3-Ha, *J*=15.5Hz), 4.80(d, 1H, 2xAll-3-Hb, *J*=13.0Hz), 4.18(s, 1H, H-1β), 4.00(dd, 1H, All-1-Ha,β, *J*=3.0Hz, *J*=9.0Hz), 3.94(d, 1H, H-1,α, *J*=8.0Hz), 3.85(dd, 1H, -All-1-Ha,α, *J*=2.8Hz, *J*=8.0Hz), 3.78(dd, 1H, All-1-Hb,β, *J*=3.0Hz, *J*=9.1Hz), 3.67(dd, 1H, All-1-Hb,α, *J*=2.9Hz, *J*=8.8Hz), 3.48(d, 1H,H-6a,α, *J*=2.7Hz), 3.43(d, 1H, H-6aβ, *J*=2.5Hz ), 3.32-3.29(m, 3H, H-4,β, H-5,β, H-6b,β), 3.20-3.10(m, 1H, H-6b,β) ,3.04(dd, 1H, H-2β, *J*=3.5Hz, *J*=6.0Hz), 3.02-2.84(m, 5H, H-2,α, H-3,α, H-4,α, H-5,α, H-3,β). 13C NMR: (500MHz, MeOD)-δ134.24(All-2-Cα), 134.10 (All-2-Cβ), 116.00(All-3-Cα), 115.99(All-3-Cβ), 101.88(C-1α), 97.70(C-1β), 76.60(C-4α), 76.45(C-4β), 77.96(C-2α) 73.61(C-2β), 72.27(C-3α), 72.04(C-3β), 70.34(All-1-Cα), 70.16(All-1-Cβ), 69.58(C-6α), 67.86(C-6β), 61.28(C-5α), 61.19(C-5β). MS electrospray (m/z) – [M+Na]+=243.1, [2M+Na]+=463.3.

**Allyl 4,6-*O*-benzylidene-α-**D**-glucopyranoside 2 [2]**

Allyl α-D-glucopyranoside (0.70g, 3.08mmol) and *para*-toluenesulphonic acid (0.06g, 0.29mmol) was dissolved in dry DMF (20ml). Benzaldehyde dimethyl acetal (1.4ml, 9.38mmol) was then added and the resulting solution stirred at 70oC for 30 hours. The solution was then poured onto ice (5g) diethyl ether (5ml) and NaHCO3 (5ml) forming a precipitate which was filtered and the remaining product then extracted with ethyl acetate (4x5ml), dried (MgSO4)and concentrated in *vacuo*. The resulting oil was then triturated with ethanol and the precipitate filtered yielding 0.81g (75%) of white crystalline solid.

[α]D20 +98.6 (c 0.023, CHCl3), (lit. [α]D20 93.8 (c 1.26, CHCl3)), 1H NMR: (500MHz, CDCl3)-δ7.50-7.48(m, 2H, Ph), 7.37-7.35(m, 3H, Ph), 5.97-5.90(m, 1H, All-2-H), 5.53(s, 1H, CHPh), 5.32(d, 1H, All-3-Ha, *J*=17.0Hz), 5.25(d, 1H, All-3-Hb, *J*=10.5Hz), 4.95(d, 1H, H-1, *J*=4.0Hz), 4.29-4.23(m, 2H, H-6b, All-1-Ha), 4.08-4.04(m, 1H, All-1-Hb), 3.96(dd, 1H, H-3, *J*=9.5Hz, *J*=2.0Hz), 3.94-3.83(m, 1H, H-5), 3.73(t, 1H, H-6a, *J*=10.5Hz), 3.63(dd, 1H, H-2, *J*=4.0Hz, *J*=2.0Hz), 3.50(t, 1H, H-4, *J*=9.5Hz). 13C NMR: (500MHz, CDCl3)-δ137.0(PhØ), 133.3(All-2-C), 129.3(Ph, 1C), 128.3(Ph, 2C), 126.3(Ph, 2C), 118.3(All-3-C), 101.9(CHPh), 97.8(C-1), 80.9(C-4), 73.2(C-2), 71.9(C-3), 68.9(C-6, All-1-C), 62.6(C-5). HRMS ES+ (m/z) [M+] found 308.1247, calculated 308.1259.

**Allyl 2,3,4,6-tetra-*O*-acetyl -β-*D*-glucopyranoside[3]**

Allyl alcohol (3.33ml, 48.0mmol) was suspended in dry DCM (20ml) with molecular sieves and to this mercury oxide (5.79g, 26.8mmol) and mercury bromide (864mg, 2.40mmol) were added in the dark. The 2,3,4,6-tetra-*O*-acetyl-α-*D-*glucopyranosyl bromide(10g, 24.3mmol) in dry DCM(10ml) was then added and the reaction mixture left to stir at room temperature overnight. Workup was then carried out by filtering through Whatman filters and washing through with DCM (10ml). The filtrate was then washed with water(10ml), NaHCO3(10ml) and brine(10ml), dried and concentrated to yield 7g (77%) of product a white solid which required no further purification.

1H NMR: (500MHz, CDCl3)-δ5.92-5.80(m, 1H, All-2-H), 5.25(d, 1H, All-3-Ha, *J*=17.5Hz), 5.22-5.11(m, 2H, H-3, All-3-Hb), 5.09(t, 1H, H-4, *J*=10Hz), 5.02(dd, 1H, H-2, *J*=8.0Hz, 9.5Hz), 4.55(d, 1H, H-1, *J*=8.0Hz), 4.33(dd, 1H, All-1-Ha, *J*=3.5Hz, 12.0Hz), 4.26(dd, 1H, H-6a, *J*=5.0Hz, 12.5Hz), 4.14(dd, 1H, H-6b, *J*=2.5Hz, 12.0Hz), 4.09(dd, 1H, All-1-Hb, *J*=6.0Hz, 12.0Hz), 3.70-3.67(m, 1H, H-5), 2.10(s, 3H, CH3), 2.05(s, 3H, CH3), 2.01(s, 3H, CH3), 2.00(s, 3H, CH3). 13C NMR: (500MHz, CDCl3)-δ171.1(PhØ), 170.7(PhØ), 169.8(PhØ), 169.7(PhØ), 133.7(All-2-C), 118.1(All-3-C), 100.0(C-1), 73.3(C-3), 72.2(C-5), 71.7(C-2), 70.4(All-1-C), 68.9(C-4), 62.4(C-6), 21.2(CH3), 21.1(CH3), 21.0(CH3). HRMS ES+ (m/z) [M+] found 388.1367, calculated 388.1369

**Allyl-β**-*D***-glucopyranoside**

Allyl 2, 3, 4, 6-tetra-*O*-acetyl-β-*D*-glucopyranoside (500mg, 1.23mmol) was dissolved in methanol (10ml) and to this sodium methoxide (133mg, 2.47mmol) was added. The reaction mixture was then left to stir at room temperature overnight. Workup was then carried out by concentrating under vacuum to yield 250mg (90%) of crude material, colourless oil that required no further purification.

1H NMR: (500MHz, CDCl3)-δ4.10-4.00(m, 1H, All-2-H), 3.43(d, 1H, All-3-Ha, *J*=17.5Hz), 3.26(d, 1H, All-3-Hb, *J*=10.5Hz), 2.47(dd, 1H, All-1-Ha, *J*=3.5Hz, 15.0Hz), 2.41(d, 1H, H-1, *J*=7.0Hz), 2.25(dd, 1H, All-1-Hb, *J*=5.0Hz, 10.0Hz), 1.97(d, 1H, H-6a, *J*=12.0Hz), 1.77(dd, 1H, H-6b, *J*=5.0Hz, 11.5Hz), 1.45-1.29(m, 4H, H-2, H-3, H-4, H-5). 13C NMR: (500MHz, CDCl3)-δ138.7 (All-2-C), 120.5(All-3-C), 106.3(C-1), 81.1(C-4), 80.9(C-5), 78.1(C-2), 74.6(C-3), 74.1(All-1-C), 65.7(C-6).

**Allyl 4,6-*O*-benzylidene-β-**D**-glucopyranoside(11) [4]**

Allyl-*β*-*D*-glucopyranoside (0.25g, 1.13mmol) and *para*-toluenesulphonic acid (0.02g, 0.11mmol) was dissolved in dry DMF (5ml). Benzaldehyde dimethyl acetal (0.83ml, 5.65mmol) was then added and the resulting solution stirred at 70oC for 16hours. The solution was then poured onto ice (5g) diethyl ether (5ml) and NaHCO3 (5ml) forming a precipitate which was filtered and the remaining product then extracted with ethyl acetate (4x5ml) dried (MgSO4) and concentrated *in vacuo*. The resulting oil was then triturated with ethanol and filtered to yield 0.3g (78%) of white crystalline solid. 1H NMR: (500MHz, CDCl3)-δ7.48-7.45(m, 2H, Ph), 7.38-7.35(m, 3H, Ph), 5.97-5.91(m, 1H, All-2-H), 5.54(s, 1H, CHPh), 5.35(d, 1H, All-3-Ha, *J*=17.0Hz), 5.25(d, 1H, All-3-Hb, *J*=10.5Hz), 4.47(d, 1H, H-1, *J*=7.5Hz), 4.40(dd, 1H, All-1-Ha, *J*=5.0Hz, 12.5Hz), 4.35(dd, 1H, H-4, *J*=5.0Hz, 10.5Hz), 4.16(dd, 1H, All-1-Hb, *J*=6.5Hz, 12.5Hz), 3.84(t, 1H, H-3, *J*=9.0Hz), 3.79(t, 1H, H6a, *J*=10.5Hz), 3.57(t, 1H, H-6b, *J*=9.5Hz), 3.55(dd, 1H, H-2, *J*=7.0Hz, 8.0Hz), 3.50-3.42(m, 1H, H-5). 13C NMR: (500MHz, CDCl3)-δ137.4(PhØ), 133.8(All-2-C), 129.7(Ph), 128.8(Ph), 126.7(Ph), 118.8(All-3-C), 102.6(C-1), 102.4(CHPh), 81.0(C-2), 75.0(C-6), 73.6(C-3), 71.1(All-1-C), 69.1(C-4), 66.9(C-5). HRMS ES+ (m/z) [M+] found 309.1343, calculated 309.1338

H

**General procedure**

Allyl 4,6-*O*-benzylidene-α-D-glucopyranoside (0.04g, 0.13mmol) was dissolved in dry solvent (0.80ml). Tetrabutylammonium iodide (0.01g, 0.02mmol) was added to this solution, followed by the alkylating, acylating or silylating reagent. Hexane-washed sodium hydride (60% in mineral oils, 0.02g, 0.56mmol) was suspended in dry solvent (0.80ml) and added to the sugar solution. The resulting solution was then left stirring at 70oC for 16hours. The reaction mixture was then allowed to cool and to this mixture a methanol/water (1:1) solution was added to destroy the remaining NaH. Brine (5ml) was then added and an extraction was carried out with DCM (4x5ml). The organic phases were then combined, washed with 10% KCl solution (2x5ml), dried and concentrated to give the crude product which was then purified by column chromatography (eluent hexane: ethyl acetate, 75:25) to yield pure product in the yields shown below.

**Allyl 2(3)-*O*-acetyl-4,6-*O*-benzylidene-α-***D***-glucopyranoside (3a ,5a) [5]**

Pure yield 0.03g (72%); Rapid acetyl migration prevented isolation and characterisation of each single regioisomer.

[α]D20 69.1 (c 0.015, CHCl3), (lit. [α]D20 131.9 (c 0.27, CHCl3)), 1H NMR: (500MHz, CDCl3)-δ7.51-7.46(m, 2H, Ph), 7.45-7.40(m, 2H, Ph), 7.38-7.33(m, 6H, Ph), 5.96-5.84(m, 2H, All-2-H, All-2-H’), 5.55(s, 1H, CHPh), 5.49(s, 1H, CHPh’), 5.37-5.20(m, 5H, H-3’, All-3-Ha,b, All-3-Ha,b’), 5.10(d, 1H, H-1, *J*=3.8Hz), 4.95(d, 1H, H-1’, *J*=3.9Hz), 4.80(dd, 1H, H-2, *J*=3.8Hz, *J*=9.7Hz), 4.28(dd, 1H, H-2’, *J*=4.9Hz, *J*=10.3Hz), 4.27-4.15(m, 3H, H-3, All-1-Ha, All-1-Ha’), 4.09-3.96(m, 2H, All-1-Hb, All-1-Hb’), 3.91-3.84(m, 2H, H-5, H-5’), 3.77-3.60(m, 4H, H-6ab, H-6ab’), 3.57(d, 1H, H-4, *J*­=9.5Hz) 3.55(d,1H, H-4’, *J*­=9.5Hz), 2.15(s, 3H, CH3), 2.13( s, 3H, CH3). 13C NMR: (500MHz, CDCl3)-δ171.10(C=O), 170.65(C=O), 137.03(PhØ), 137.00(PhØ), 133.38(All-2-C), 133.19(All-2-C), 129.30, 129.09, 128.37, 128.24, 126.32, 126.26(Ph), 118.46(All-3-C), 117.91(All-3-C), 102.07(CHPh), 101.55(CHPh), 98.28(C-1), 95.70(C-1’), 81.46(C-6), 78.71(C-6’), 73.64(C-2’), 72.36(C-3), 71.81(C-4), 70.75(C-4’), 69.05(C-3’), 68.88(All-1-C, C-2), 68.68(All-1-C’), 62.97(C-5), 62.2(C-5’). HRMS ES+ (m/z) [M+] found 350.1362, calculated 350.1365.

**Allyl 2,3-di-*O*-acetyl-4,6-*O*-benzylidene-α-***D***- glucopyranoside (6a) [6]**

Pure yield 0.04g (75%)

1H NMR: (500MHz, CDCl3)-δ7.45-7.439m, 2H, Ph), 7.36-7.34(m, 3H, Ph), 5.91-5.83(m, 1H, All-2-H), 5.62(t, 1H, H-3, *J*=9.8Hz), 5.51(s, 1H, CHPh), 5.33(d, 1H, All-3-Ha, *J*=1.6Hz), 5.30-5.21(m, 1H, All-3-Hb), 5.11(d, 1H, H-1, *J*=3.7Hz), 4.90(dd, 1H, H-2, *J*=3.8Hz, *J*=9.9Hz), 4.30-4.23(m, 1H, All-1-Ha), 4.20-4.18(m, 1H, All-1-Hb), 4.04-3.97(m, 1H, H-5), 3.78-3.76(m, 1H, H-6a), 3.75(t, 1H, H-4, *J*=10.3Hz), 3.66(t, 1H, H-6b, *J*=9.6Hz), 2.09-2.03(m, 6H, 2CH3) 13C NMR: (500MHz, CDCl3)-δ171.44(C=O), 170.2(C=O), 133.28(All-2-C), 129.07(Ph, 2C), 128.24(Ph, 2C), 126.16(Ph, 1C), 118.01(All-3-C), 101.58(CHPh), 95.73(C-1), 79.36(C-6), 71.61(C-2), 69.06(C-4), 68.85(C-3), 68.73(All-1-C), 62.57(C-5), 20.88(CH3), 20.70(CH3). HRMS ES+ (m/z) [M+] found 392.1462, calculated 392.1471.

**Allyl 2(3)-*O*-allyl-4,6-*O*-benzylidene-α-***D***- glucopyranoside (3b,5b)**

Pure yield 0.03g (62%)

1H NMR: (500MHz, CDCl3)-δ7.51-7.47(m, 2H, Ph), 7.38-7.32(m, 3H, Ph), 5.99-5.89(m, 2H, All-2-H), 5.53(d, 1H, CHPh, *J*=3.6Hz), 5.37-5.16(m, 4H, All-3-H2), 4.99(d, 1H, H-1, *J*=3.6Hz), 4.95(d, 1H, H-1’, *J*=3.8Hz), 4.42(dd, 1H, All-1-Ha,*J*=5.5Hz, *J*=12.7Hz), 4.28-4.24(m, 4H, All-1-Hb, H-3, All-1-Ha,b’), 4.23-4.21(m, 2H, H-3’, All-1-Ha’), 4.19-4.18(m, 2H, All-1-Hb’, H-6a), 4.10(dd, 1H, H-6b, *J*=4.8Hz, *J*=11.3Hz), 3.89-3.77(m, 2H, H-5, H-5’), 3.75-3.68(m, 2H, H-2, H-6a), 3.68-3.66(m, 1H, H-6b), 3.58(dd, 1H, H-4, *J*=9.25Hz, *J*=9.30Hz), 3.53(dd, 1H, H-4’, *J*=9.40Hz, *J*=9.45Hz), 3.44(dd, 1H, H-2, *J*=3.6Hz, *J*=9.3Hz) 13C NMR: (500MHz, CDCl3)-δ137.36, 137.11(PhØ), 135.03, 134.56(All-2-C), 133.55, 133.40(All-2-C), 129.20, 128,95(Ph, 2C), 128.31, 128.24(Ph, 4C), 126.35, 126.00,(Ph, 4C), 118.25, 118.05, 117.22(All-3-C), 102.0, 101.25(CHPh), 98.00, 96.16(C-1), 81.95, 81.35(C-4), 79.45, 78.57(C-2), 73.76(C-6), 72.27, 72.03(All-1-C), 70.08(C-3), 69.02(C-6), 68.96, 68.80(All-1-C), 68.53(C-3), 62.82, 62.34(C-5). HRMS ES+ (m/z) [M+] found 348.1568, calculated 348.1572.

**Allyl 2,3-di-*O*-allyl-4,6-*O*-benzylidene-α-***D***- glucopyranoside (6b) [7]**

Pure yield 0.03g (64%)

1H NMR: (500MHz, CDCl3)-δ7.49-7.48(m, 2H, Ph), 7.37-7.34(m, 3H, Ph), 5.98-5.90(m, 3H, All-2-H), 5.53(s, 1H, CHPh), 5.36-5.12(m, 6H, All-3-H2), 4.93(d, 1H, H-1, *J*=4.00Hz), 4.39-4.35(m, 1H, All-1-Ha), 4.29-4.14(m, 7H, 2All-1-Hb, 3All-1-Hb, H-6a), 3.91-3.84(m, 2H, H-5,H-6b), 3.70(t, 1H, H-4, *J*=10.5Hz), 3.54(dd, 1H, H-3, *J*=9.5Hz, *J*=5.0Hz), 3.46(dd, 1H, H-2, *J*=4.0Hz, *J*=9.5Hz). 13C NMR: (500MHz, CDCl3)-δ136.4(PhØ), 134.2(All-2-C), 133.8(All-2-C), 132.6(All-2-C), 127.8(Ph, 1C), 127.1(Ph, 2C), 125.0(Ph, 2C), 117.2, 116.4, 115.5(All-3-C), 100.2(CHPh), 95.8(C-1), 81.1(C-3), 78.0(C-2), 77.0(All-1-C), 72.97(C-6), 71.8(Al-1-C), 68.0(C-4), 67.3(All-1-C), 61.5(C-5). HRMS ES+ (m/z) [M+] found 388.1897, calculated 388.1885.

**Allyl 2-*O*-benzyl-4,6-*O*- benzylidene-α-***D***- glucopyranoside(3d)[8]**

Pure yield 0.04g (68%)

[α]D20 11.0 (c 0.002, CHCl3), 1H NMR: (500MHz, CDCl3)-δ7.50-7.49(m, 2H, Ph), 7.48-7.34(m, 8H, Ph), 5.96-5.88(m, 1H, All-2-H), 5.54(s, 1H, CHPh), 5.35(d, 1H, All-3-Ha, *J*=1.5Hz), 5.32(d, 1H, All-3-Hb, *J*=1.5Hz), 4.81(d, 1H, H-1, *J*=3.7Hz), 4.76-4.67( AB, 2H, CH2Ph, *J*a=12.1Hz, *J*b=12.0Hz, δν=38.7Hz), 4.24(dd, 1H, H-6a, *J*=4.9Hz, J=10.2Hz), 4.21-4.15(m, 2H, H-3, All-1-Ha), 3.97(dd, 1H, All-1-Hb, *J*=6.5Hz, *J*=12.9Hz), 3.90-3.85(m, 1H, H-5), 3.70(t, 1H, H-6b, *J*=10.3Hz), 3.51(dd, 1H, H-4, *J*=9.4Hz, *J*=9.3Hz), 3.47(dd, 1H, H-2, *J*=3.6Hz, *J*=9.2Hz). 13C NMR: (500MHz, CDCl3)-δ137.9(PhØ), 133.5(All-2-C), 129.1(Ph, 1C), 128.5(Ph, 2C), 128.3(Ph,2C) 128.1(Ph, 2C),127.9(Ph, 1C), 126.3(Ph, 2C), 118.2(All-3-C), 101.9(CHPh), 96.3(C-1), 81.3(C-4), 79.6(C-2), 73.1(CH2Ph), 70.1(C-3), 68.9(All-1-C), 68.6(C-6), 62.3(C-5). HRMS ES+ (m/z) [M+] found 398.1718, calculated 398.1729.

**Allyl 4,6-*O*-benzylidene-2(3)-*O*-propargyl-α-***D***-glucopyranoside(3c, 5c).**

Pure yield 0.04g (68%)

1H NMR: (500MHz, CDCl3)-δ7.50-7.46(m, 4H, Ph), 7.36-7.26(m, 4H, Ph), 5.97-5.92(m, 2H, All-2-H, All-2-H’)), 5.54(s, 1H, CHPh), 5.53(s, 1H, CHPh’), 5.33(dd, 2H, All-1Ha, All-1-Ha’, *J*=5.0Hz, 10.0Hz), 5.25(dd, 2H, All-1-Hb, All-1-Hb’, *J*=1.5Hz, 12.0Hz), 5.09(d, 1H, H-1, *J*=3.5Hz), 4.97(d, 1H, H-1’, *J=*4.0Hz), 4.55-4.32(m, 4H, PrH), 4.30-4.18(m, 2H, H-4, H-4’), 4.17-4.02(m, 6H, All-3-Ha, All-3-Hb, All-3-Ha’, All-3-Hb’, H-3, H-3’), 3.96-3.92(m, 2H, H-5, H-5’), 4.78(dd, 2H, H-6a, H-6a’, *J*=10.0Hz, 9.5Hz), 4.74(dd, 2H, H-2, H-2’, *J*=2.5Hz, 9.5Hz), 4.60(dd, 1H, H-6b, *J*=10.0Hz, 9.5Hz), 4.57(dd, 1H, H-6b’, *J*=9.0Hz, 10.0Hz), 2.42(m, 2H, PrH). 13C NMR: (500MHz, CDCl3)-δ138.76(PhØ), 138.75(PhØ), 133.91(All-2-C), 133.78(All-2-C’), 129.44(Ph), 128.75(Ph), 128.67(Ph), 126.74(Ph), 126.46(Ph), 118.72(All-1-C), 118.68(All-1-C’), 102.44(CHPh), 101.75(CHPh’), 98.38(C-1), 96.76(C-1’), 82.16(C-6), 81.24(C-6’), 79.23(CPr), 78.57(CPr’), 75.70(C-4), 75.12(C-4’), 72.34(C-2), 70.60(C-3), 69.40(C-2’), 69.26(C-3’), 69.12(C-5), 69.07(C-5’), 63.10(PrC), 62.70(PrC).

**Allyl 4,6-*O*-benzylidene-2,3-di-*O*-propargyl-α-***D***-glucopyranoside(6c).**

Pure yield=0.04g (75%)

1H NMR: (500MHz, CDCl3)-δ7.43-7.41(m, 2H, Ph), 7.37-7.34(m, 3H, Ph), 6.00-5.90(m, 1H, All-2-H), 5.54(s, 1H, CHPh), 5.34(d, 1H, All-3-Ha, *J*=17.0Hz), 5.23(d, 1H, All-3-Hb, *J*=10.5Hz), 5.03(d, 1H, H-1, *J*=3.5Hz), 4.47-4.42(m, 4H, CH2Pr), 4.26(dd, 1H, H-4, *J*=5.5Hz, 10.5Hz), 4.20(dd, 1H, All-1-Ha, *J*=6.5Hz, 11.5Hz), 4.11(dd, 1H, All-1-Hb, *J*=6.5Hz, 12.0Hz), 4.00(dd, 1H, H-3, *J*=9.5Hz, 10.0Hz), 3.94-3.84(m, 1H, H-5), 3.75(dd, 1H, H-6a, *J*=9.0Hz, 9.5Hz), 3.72(dd, 1H, H-2, *J*=5.5Hz, 8.5Hz), 3.62(dd, 1H, H-6b, *J*=9.0Hz, 9.5Hz), 2.45-2.43(m, 2H, Pr). 13C NMR: (500MHz, CDCl3)-δ137.67(PhØ), 133.97(All-2-C), 129.40(Ph), 128.64(Ph), 126.48(Ph), 118.66(All-1-C), 103.01(CHPh), 101.64(C-1), 82.47(C-6), 80.47(All-3-C), 78.41(C-3), 75.21(PrC), 74.70(PrC), 69.42(C-2), 69.04(C-4), 62.79(C-5), 60.50(PrC), 59.64(PrC).

**Allyl 2,3-di-*O*-benzyl-4,6- *O*-benzylidene-α-***D****-* glucopyranoside (6d) [9]**

Pure yield 0.04g (66%)

1H NMR: (500MHz, CDCl3)-δ7.49-7.48(m, 1H, Ph), 7.39-7.26(m, 13H, Ph), 5.98-5.90(m, 1H, All-2-H), 5.55(s, 1H, CHPh), 5.33(d, 1H, All-3-Ha, *J*=15.5Hz), 5.23(d, 1H, All-3-Hb, *J*=10.0Hz), 4.91(d, 1H, All-1-Ha, *J*=11.0Hz), 4.85-4.81(m, 2H, CH2Ph), 4.79(d, 1H, H-1, *J*=3.5Hz), 4.68(d, 1H, All-1-Hb, *J*=12.5Hz), 4.25(dd, 1H, H-5, *J*=5.0Hz, *J*=10.0Hz), 4.18(dd, 1H, H-4, *J*=5.0Hz, *J*=13.0Hz), 4.09-4.01(AB, 2H, CH2Ph *J*a=9.3Hz, *J*b=6.13Hz, δν=22.7Hz), 3.91-3.86(m, 1H, H-3), 3.69(t, 1H, H-6a, *J*=10.5Hz), 3.60(dd, 1H, H-6b, *J*=9.0Hz, *J*=9.5Hz), 3.56(dd, 1H, H-2, *J*=3.8Hz, *J*=9.3Hz)

13C NMR: (500MHz, CDCl3)-δ138.18, 138.23, 137.43(PhØ), 133.64(ALL-2-C), 128.90(Ph, 1C), 128.41(Ph, 2C), 128.29(Ph, 2C), 128.25(Ph, 2C), 128.06(Ph, 2C), 127.99(Ph, 2C), 127.86(Ph, 1C), 127.56(Ph, 1C), 126.01(Ph, 2C), 118.35(All-3-C), 101.25(CHPh), 96.80(C-1), 82.23(C-6), 79.27(C-2), 78.64(CH2Ph), 75.36(All-1-C), 73.61(CH2Ph), 69.04(C-4), 68.52(C-5), 62.56(C-3). HRMS ES+ (m/z) [M+] found 488.2198, calculated 488.2198

**Allyl 2-O-benzoyl-4,6-O-benzylidene-α-***D***- glucopyranoside (3e)[10]**

Pure yield 0.03g (57%)

[α]D20 75.8 (c 0.011, CHCl3), (lit. [α]D20 99 (c 1, CHCl3)), 1H NMR: (500MHz, CDCl3)-δ8.02-8.00(d, 2H, Ph, J=8.34Hz), 7.52-7.28(m, 8H, Ph), 5.78-5.71(m, 1H, All-2-H), 5.50(s, 1H, CHPh), 5.22-5.18(d, 1H, All-3-Ha, *J*=18.8Hz), 5.14-5.13(d, 1H, H-1, *J*=3.8Hz), 5.08-5.05(d, 1H, All-3-Hb, *J*=10.4Hz), 5.00-4.97(dd, 1H, H-2, *J*=3.8Hz, *J*=9.7Hz), 4.33-4.30(dd, 1H, H-3, *J*=4.2Hz, *J*=9.4Hz), 4.25-4.22(dd, 1H, H-4, *J*=4.9Hz, *J*=10.5Hz), 4.15-4.11(dd, 1H, All-1-Ha, *J*=5.1Hz, *J*=13.4Hz), 3.95-3.87(m, 2H, All-1-Hb, H-5), 3.73-3.69(dd, 1H, H-6a, *J*=10.4Hz, *J*=10.3Hz), 3.58-3.54(t, 1H, H-6b, *J*=9.4Hz) 13C NMR: (500MHz, CDCl3)-δ165.20(C=O), 136.00(PhØ), 132.36(PhØ), 132.33(All-2-C), 128.88(Ph, 2C), 128.54(Ph), 128.27(Ph), 127.42(Ph, 2C), 127.33(Ph, 2C), 125.30(Ph, 2C), 116.67(All-3-C), 101.04(CHPh), 94.96(C-1), 80.50(C-6), 72.97(C-2), 67.87(C-3), 67.80(C-4), 67.77(All-1-C), 61.31(C-5) HRMS ES+ (m/z) [M+] found 412.1540, calculated 412.1522.

**Allyl 2,3-di-*O*-benzoyl-4,6-*O*-benzylidene-α-***D***- glucopyranoside (6e) [11]**

Pure yield 0.04g (62%)

[α]D20 45.6 (c 0.017, CHCl3)) (lit. [α]D20 39 (c 1, CHCl3)), 1H NMR: (500MHz, CDCl3)-δ8.09-7.89(m, 4H, Ph), 7.55-7.22(m, 11H, Ph), 6.01(dd, 1H, H-2, *J*=9.8Hz, *J*=9.7Hz), 5.78-5.73(m, 1H, All-2-H), 5.49(s, 1H, CHPh), 5.23(d, 1H, H-1, *J*=3.7Hz), 5.20-5.18(m, 1H, All-3-Ha), 5.08-5.04(m, 1H, H-1, All-3-Hb), 4.28(dd, 1H, H-5, *J*=4.8Hz, *J*=10.3Hz), 4.16-4.15(m, 1H, All-1-Ha) , 4.07-4.00(m, 1H, H-4), 3.99-3.95(m, 1H, All-1-Hb), 3.83(t, 1H, H-3, *J*=9.6Hz), 3.77(dd, 1H, H-6a, *J*=4.7Hz, *J*=10.3Hz), 3.73(dd, 1H, H-6b, *J*=4.7Hz, *J*=11.1Hz). 13C NMR: (500MHz, CDCl3)-δ170.40(2xC=O), 132.72(2xPh, All-2-C), 129.17(Ph, 2C), 128.89(Ph, 2C), 128.71(Ph, 2C), 127.46(Ph,2C), 127.25(Ph, 3C), 127.15(Ph, 2C), 125.13(Ph, 3C), 116.21(All-3-C), 102.35(CHPh), 95.12(C-1), 78.23(C-6), 72.20(All-1-C), 70.90(C-2), 68.91(All-2-C), 67.24(C-3), 66.87(C-5), 62.85(C-4).

HRMS ES+ (m/z) [M+] found 516.1807, calculated 516.1784.

**Allyl 4,6-*O*-benzylidene-2-*O*-*para* methoxybenzyl-α-***D* **glucopyranoside (3f)**

Pure yield 0.03g (56%)

1H NMR: (500MHz, CDCl3)-δ7.42-7.41(m, 1H, PMB-H), 7.30-7.27(m, 5H, Ph), 6.81(d, 2H, PMB-H, *J*=8.5Hz), 5.90-5.81(m, 1H, All-2-H), 5.44(s, 1H, CHPh), 5.26(d, 1H, All-3-Ha, *J*=15.9Hz), 5.16(d, 1H, All-3-Hb, *J*=10.3Hz), 4.71(d, 1H, H-1, *J*=3.6Hz), 4.57(AB, 2H, CH2Ph, *J*a=11.7Hz, *J*b=11.8Hz, δν=33.0Hz), 4.17(dd, 1H, All-1-Ha, *J*=4.8Hz, *J*=10.2Hz), 4.11-4.08(m, 2H, H-3, All-1-Hb), 3.91(dd, 1H, H-4, *J*=6.4Hz, *J*=12.8Hz), 3.82-3.74(m, 1H, H-5), 3.73(s, 3H, CH3), 3.62(dd, 1H, H-6a, *J*=10.3Hz, *J*=10.4Hz), 3.44(t, 1H, H-6b, *J*=9.4Hz), 3.37(dd, 1H, H-2, *J*=3.6Hz, *J*=9.2Hz). 13C NMR: (500MHz, CDCl3)-δ158.55(PMB-O- PhØ), 136.11(PhØ), 132.58(All-2-C), 128.95(PMB- PhØ), 128.71(Ph, 2C), 128.15(Ph 1C), 127.27(Ph, 2C), 125.32(Ph, 2C), 117.13(All-3-C), 112.97(Ph, 2C), 100.94(CHPh), 95.36(C-1), 80.32(C-6), 78.28(C-2), 71.71(CH2Ph), 69.08(C-3), 67.97(C-4), 67.61(All-1-C), 61.29(C-5), 54.28(CH3). HRMS ES+ (m/z) [M+NH4] found 446.2176, calculated 446.2173

**Allyl 4,6-*O*-benzylidene-2,3-di-*O*-*para* methoxybenzyl-α-***D***- glucopyranoside (6f)**

Pure yield 0.04g (53%)

[α]D20 -18.1 (c 0.005, CHCl3), 1H NMR: (500MHz, CDCl3)-δ7.49-7.40(m, 2H, PMB-H), 7.39-7.36(m, 3H, PMB-H, Ph), 7.35-7.25(m, 4H, Ph), 6.89-6.81(m, 4H, PMB-H), 5.97-5.90(m, 1H, All-2-H), 5.55(s, 1H, CHPh) 5,32(d, 1H, All-3-Ha, *J*=15.6Hz), 5.23(d, 1H, All-3-Hb, *J*=11.8Hz), 4.82(d, 1H, All-1-Ha, *J*=3.0Hz), 4.76(d, 1H, CH2Pha, *J*=10.8Hz), 4.74(d, 1H, All-1-Hb, *J*=6.1Hz), 4.72(d, 1H, H-1, *J*=3.7Hz), 4.61(d, 1H, CH2Phb, *J*=11.7Hz), 4.24(dd, 1H, H-4, *J*=4.8Hz, *J*=10.1Hz), 4.16(dd, 1H, CHPha, *J*=5.1Hz, *J*=12.8Hz), 4.11(AB, 2H, CH2PhOMe *J*a=7.1Hz, *J*b=7.2Hz, δν=20.1Hz), 4.03(m, 2H, CH2PhOMe, H-3), 3.89-3.84(m, 1H, H-5), 3.80(s, 3H, OMe), 3.78(s, 3H, OMe), 3.68(dd, 1H, H-6a, *J*=10.2Hz, *J*=10.3Hz), 3.57(dd, 1H, H-6b, J=9.4Hz, *J*=9.5Hz), 3.52(dd, 1H, H-2, *J*=3.7Hz, *J*=9.3Hz). 13C NMR: (500MHz, CDCl3)-δ159.39(PMB-O- PhØ), 159.19(PMB-O- PhØ), 137.49(PhØ), 133.71(All-2-C), 131.05(PMB- PhØ), 130.36(PMB- PhØ), 129.66(Ph, 2C), 129.64(Ph, 1C), 128.87(Ph, 2C), 128.62(Ph, 1C), 128.20(Ph, 2C), 126.04(Ph, 2C), 118.26(All-3-C), 113.98(Ph, 2C), 113.82(Ph, 2C), 101.23(CHPh), 96.9(C-1), 82.81(C-6), 78.86(C-2), 78.35(CH2PMB), 75.04(All-1-C), 73.22(C-3), 69.08(CH2PMB), 68.50(C-4), 60.38(C-5), 55.27(2CH3).

HRMS ES+ (m/z) [M+] found 548.2406, calculated 548.2410.

**Allyl 4,6-*O*-benzylidene-2-*O*-*tert-*butyldiphenylsilyl –α-***D***-glucopyranoside (3f)**

Pure yield 0.03g (45%)

[α]D20 22.6 (c 0.002, CHCl3), 1H NMR: (500MHz, CDCl3)-7.60-7.00(m, 15h, Ph), 5.83-5.75(m, 1H, All-2-H), 5.18(s, 1H, CHPh), 5.08(dd, 2H, All-3-Ha,b, *J*=15.0Hz, *J*=10.0Hz), 4.82(d, 1H, H-1, *J*=4.9Hz), 4.13(dd, 1H, H-4, *J*=9.6Hz, *J*=3.8Hz), 4.07(dd, 1H, All-1Ha, *J*=4.2Hz, *J*=11.0Hz), 3.94(dd, 1H, H-3, *J*=9.6Hz, *J*=9.9Hz), 3.87(dd, 1H, All-1-Hb, *J*=4.8Hz, *J*=10.2Hz), 3.74-3.60(m, 2H, H-2, H-5), 3.55(dd, 1H, H-6a, *J*=8.9Hz, *J*=8.5zHz), 3.43(dd, 1H, H-6b, *J*=15.0Hz, *J*=10.2Hz ), 0.95(s, 9H, tBut). 13C NMR: (500MHz, CDCl3)-δ136.0, 135.19, 134.85(PhØ), 132.5(All-2-C), 128.46, 127.65, 126.80, 126.30, 126.20, 125.20(15C, Ph), 116.62(All-3-C), 100.48(CHPh), 97.17(C-1), 80.49(C-6), 76.80(C-3), 67.83(C-2), 67.46(All-1-C), 61.66(C-4), 61.11(C-5), 25.96(tBut). HRMS ES+ (m/z) [M+] found 546.2436, calculated 546.2437.

**Allyl 4,6-*O*-benzylidene-2-*O*-trimethylsilyl-α-***D-***glucopyranoside (3g)**

Pure yield 0.03g (50%)

[α]D20 51.7 (c 0.005, CHCl3), 1H NMR: (500MHz, CDCl3)-7.36-7.35(m, 2H, Ph), 7.24-7.20(m, 3H, Ph), 5.85-5.76(m, 1H, All-2-H), 5.38(s, 1H, CHPh), 5.20(d, 1H, All-3-Ha, *J*=17.2Hz), 5.09(d, 1H, All-3-Hb, *J*=10.6Hz), 4.83(d, 1H, H-1, *J*=3.9Hz), 4.14-4.10(m, 2H, All-1-Ha, H-6a), 3.95(dd, 1H, All-1-Hb, *J*=6.4Hz, *J*=16.4Hz), 3.77(dd, 1H, H-3, *J*=9.0Hz, J=9.1Hz), 3.75-3.65(m, 1H, H-5), 3.58(dd, 1H, H-6b, *J*=10.2Hz, *J*=10.3Hz), 3.33(dt, 1H, H-2, *J*=3.9Hz, *J*=8.9Hz), 3.28(dd, 1H, H-4, *J*=9.4Hz, *J*=9.3Hz), 0.00(s, 9H, 3CH3). 13C NMR: (500MHz, CDCl3)-δ136.84(PhØ), 132.97(All-2-C), 128.39, 127.62, 125.77, 125.63(Ph), 117.69(All-3-C), 101.08(CHPh), 97.67(C-1), 80.96(C-4), 72.88(C-2), 72.36(C-3), 68.41(C-6), 68.27(All-2-C), 62.42(C-5). HRMS ES+ (m/z) [M+] found 380.1649, calculated 380.1655.

**Allyl 3-*O*-acetyl -2-*O*-benzyl-4,6-*O*-benzylidene-α-***D***-glucopyranoside (4)**

Allyl 2-O-benzyl-4, 6-O-benzylidene-α-*D*-glucopyranoside (0.02g, 0.05mmol) was dissolved in dry pyridine (2ml) in a microwave flask followed by the addition of DMAP (0.001g, 0.005mmol) and acetic anhydride (15μl, 0.15mmol). This was then sealed and microwaved at 70°C and 50W for 5 minutes. Work up was carried out by addition of DCM (10ml) concentrated in vacuo followed by addition of water (10ml) and extraction with DCM (3x10ml), the organic phases were combined, dried and concentrated to give 0.02g (80%) of crude product a yellowish syrup which required no further purification.

[α]D20 55.0 (c 0.002, CHCl3), 1H NMR: (500MHz, CDCl3)-δ7.37-7.34(m, 2H, Ph), 7.29-7.22(m, 8H, Ph), 5.88-5.81(m, 1H, All-2-H), 5.51(t, 1H, H-3, *J*=9.5Hz), 5.38(s, 1H, CHPh), 5.26(d, 1H, All-3-Ha, *J*=17.0Hz), 5.15(d, 1H, All-3-Hb, *J*=10.5Hz), 4.79(d, 1H, H-1, *J*=3.5Hz), 4.55(d, 2H, CH2Ph, *J*=2.0Hz), 4.17(dd, 1H, H-6a, *J*=10.5Hz, *J*=5.5Hz), 4.10(d, 1H, All-1-Ha, *J*=8.0Hz), 3.94(d, 1H, All-1-Hb, *J*=6.5Hz), 3.86(dt, 1H, H-5, *J*=4.5Hz, *J*=10.0Hz), 3.61(dd, 1H, H-6b, *J*=6.0Hz, *J*=10.5Hz), 3.50(dd, 1H, H-2, *J*=3.4Hz, *J*=9.5Hz), 3.46(t, 1H, H-4, *J*=9.5Hz), 1.97(s, 3H, AcCH3) 13C NMR: (500MHz, CDCl3)-δ168.7(C=0), 136.8(PhØ), 136.0(PhØ), 132.4(All-2-C), 127.9(Ph, 1C), 127.4(Ph, 2C), 127.1(Ph, 2C), 126.9(Ph, 2C), 126.8(Ph, 2C), 125.1(Ph, 1C), 117.2(All-3-C), 100.4(CHPh), 95.4(C-1), 78.6(C-4), 76.5(C-2), 71.7(CH2Ph), 69.5(C-3), 67.9(C-6), 67.6(All-1-C), 61.5(C-5), 19.9(CH3). HRMS ES+ (m/z) [M+] found 440.1829, calculated 440.1835.

**Allyl -2,3di-*O*-benzyl-4,6-*O*-benzylidene-β**-*D***-glucopyranoside (14)**

Synthesis as for general synthetic procedure

Pure yield=0.03g (70%)

1H NMR: (500MHz, CDCl3)-δ7.50-7.46(m, 3H, Ph), 7.34-7.26(m, 12H, Ph), 5.98-5.93(m, 1H, All-2-H), 5.57(s, 1H, CHPh), 5.35(d, 1H, All-3-Ha, *J*=17.5Hz), 5.23(d, 1H, All-3-Hb, *J*=10.5Hz), 4.91(d, 2H, CH2Ph, *J*=11.5Hz), 4.79(t, 2H, CH2Ph, *J*=13.5Hz), 4.56(d, 1H, H-1, *J*=8.0Hz), 4.41(dd, 1H, All-1-Ha, *J*=5.0Hz, 12.5Hz), 4.35(dd, 1H, H-4, *J*=5.0Hz, 10.5Hz), 4.16(dd, 1H, All-1-Hb, *J*=6.0Hz, 12.5Hz), 3.79(t, 1H, H-3, *J*=10.5Hz), 3.74(t, 1H, H-6a, *J*=9.0Hz), 3.69(t, 1H, H-6b, *J*=8.5Hz), 3.50(t, 1H, H-2, *J*=8.5Hz), 3.44-3.38(m, 1H, H-5). 13C NMR: (500MHz, CDCl3)-δ135.9(PhØ), 138.8(PhØ), 137.8(PhØ), 134.2(All-2-C), 129.6(Ph), 128.8(Ph), 128.7(Ph), 128.6(Ph), 128.5(Ph), 128.4(Ph), 128.1(Ph), 128.0(Ph), 126.4(Ph), 118.0(All-3-C), 103.6(CHPh), 101.6(C-1), 82.6(C-2), 81.9(C-6), 81.3(C-3), 75.8(CH2Ph), 75.6(CH2Ph), 71.2(All-1-C), 69.2(C-4), 66.5(C-5). HRMS ES+ (m/z) [M+] found 488.2198, calculated 488.2198

**Methyl-4,6-benzylidene-α-***D***-glucopyranoside(15)[12]**

Methyl-*α*-*D*-glucopyranoside (5.0g, 29.4mmol) and *para*-toluenesulphonic acid (0.559g, 2.94mmol) was dissolved in dry DMF (20ml). Benzaldehyde dimethyl acetal (20.0ml, 147.0mmol) was then added and the resulting solution stirred at 70oC for 16hours. The solution was then poured onto ice (5g) diethyl ether (5ml) and NaHCO3 (5ml) forming a precipitate which was filtered and the remaining product then extracted with ethyl acetate (4x5ml) dried (MgSO4) ) and concentrated *in vacuo*. The resulting oil was then triturated with ethanol to yield 6.0g (70%) of white crystalline solid.

[α]D20 73.7 (c 0.03, CHCl3), 1H NMR: (500MHz, CDCl3)-δ7.50-7.49(m, 2H, Ph), 7.38-7.36(m, 3H, Ph), 5.54(s, 1H, CHPh), 4.82(d, 1H, H-1, *J*=3.5Hz), 4.31(dd, 1H, H-6a, *J*=4.5Hz, 9.5Hz), 3.94(dt, 1H, H-3, *J*=2.0Hz, 7.5Hz), 3.82(dd, 1H, H-5, *J*=4.0Hz, 10.0Hz), 3.75(t, 1H, H-6b, *J*=10.0Hz), 3.64(dt, 1H, H-2, *J*=4.8Hz), 3.50(t, 1H, H-4, *J*=9.5Hz), 3.47(s, 3H, CH­­3). 13C NMR: (500MHz, CDCl3)-δ137.8(PhØ), 129.7(Ph), 128.8(Ph), 126.7(Ph), 102.4(CHPh), 100.2(C-1), 81.3(C-4), 73.4(C-2), 72.3(C-3), 69.4(C-6), 62.8(C-5), 56.0(CH3).

HRMS ES+ (m/z) [M+] found 283.1183, calculated 283.1181.

**Methyl 2,3-di-*O*-benzyl-4,6-*O*-benzylidene -α-***D***-glucopyranoside(19a) [13]**

Synthesis as for the general synthetic procedure.

Pure yield=0.03g (66%)

1H NMR: (500MHz, CDCl3)-δ7.45-7.42(m, 2H, Ph), 7.39-7.26(m, 13H, Ph), 5.55(s, 1H, CHPh), 4.91(d, 1H, CH2Ph, *J*=11Hz, 5Hz), 4.84(AB, 2H, CH2Ph, *J*a=9.1Hz, *J*b=6.5Hz, δv=34.6Hz), 4.70(d, 1H, CH2Ph, *J*=12.5Hz), 4.60(d, 1H, H-1, *J*=3.5Hz), 4.26(dd, 1H, H-6a, *J*=5.0Hz, 10.0Hz), 4.05(t, 1H, H-3, *J*=9.5Hz), 3.82(dt, 1H, H-5, *J*=5.0Hz, 10.0Hz), 3.71(t, 1H, H-6b, *J*=10.0Hz), 3.60(t, 1H, H-4, *J*=9.5Hz), 3.56(dd, 1H, H-2, *J*=4.0Hz, 9.5Hz), 3.40(s, 3H, CH3). 13C NMR: (500MHz, CDCl3)-δ139.0(PhØ), 138.6(PhØ), 137.8(PhØ), 129.3(Ph), 128.8(Ph), 128.7(Ph), 128.6(Ph), 128.5(Ph), 128.4(Ph), 128.3(Ph), 127.9(Ph), 126.5(Ph), 101.7(CHPh), 99.7(C-1), 82.6(C-4), 79.6(C-2), 79.0(C-3), 75.8(CH2Ph), 74.2(CH2Ph), 69.5(C-6), 62.8(C-5), 55.9(CH3).

**Benzyl 4,6-*O*-benzylidene-α-***D***-glucopyranoside (16) [14]**

*D*-Glucose (5.0g, 27.7mmol) was partially dissolved in benzyl alcohol (10ml). To this suspension *para*-toluenesulphonic acid (0.53g, 2.77mmol) was then added and the resulting suspension stirred for 16hours at 90oC. It was then concentrated *in vac uo* to give crude1-*O*-benzyl-*α*-*D*-glucopyranoside. The Benzyl *α*-*D*-glucopyranoside (7.0g, 25.9mmol) and *para*-toluenesulphonic acid (0.49g, 2.59mmol) was dissolved in dry DMF (20ml). Benzaldehyde dimethyl acetal (19.0ml, 129.6mmol) was then added and the resulting solution stirred at 70oC for 16hours. The solution was then poured onto ice (5g) diethyl ether (5ml) and NaHCO3 (5ml) forming a precipitate which was filtered and the remaining product then extracted with ethyl acetate (4x5ml) dried (MgSO4) and concentrated *in vacuo*. The resulting oil was then triturated with ethanol and filtered to yield 5.0g (49%) of white crystalline solid.

[α]D20 107.3 (c 0.07, CHCl3)), (lit. [α]D20 106.4 (c 1, CHCl3)), 1H NMR: (500MHz, CDCl3)-δ7.50-7.48(m, 2H, Ph), 7.38-7.33(m, 8H, Ph), 5.53(s, 1H, CHPh), 5.03(d, 1H, H-1, *J*=4.0Hz), 4.78(d, 1H, CH2Ph, *J*=11.5Hz), 4.58(d, 1H, CH2Ph, *J*=12.0Hz), 4.24(dd, 1H, H-6a, *J*=5.0Hz, 10.0Hz), 3.97(t, 1H, H-3, *J*=9.0Hz), 3.87(dt, 1H, H-5, *J*=5.0Hz, 10.5Hz), 3.74(t, 1H, H-2, *J*=10.0Hz), 3.70(dd, 1H, H-6b, *J*=3.5Hz, 10.5Hz), 3.52(t, 1H, H-4, *J*=9.5Hz). 13C NMR: (500MHz, CDCl3)-δ137.4(PhØ), 137.1(PhØ), 129.7(Ph), 129.1(Ph), 128.8(Ph), 128.7(Ph), 128.6(Ph), 126.7(Ph), 102.3(CHPh), 98.6(C-1), 81.3(C-4), 73.4(C-2), 72.4(C-3), 70.7(CH2Ph), 69.3(C-6), 63.2(C-5). HRMS ES+ (m/z) [M+] found 359.1488, calculated 359.1494

**Benzyl 2,3-di-*O*-benzyl-4,6-*O*-benzylidene-α-***D-***glucopyranoside(19b)[15]**

Synthesis as for general synthetic procedure

Pure yield=0.04g(70%)

1H NMR: (500MHz, CDCl3)-δ7.50-7.47(m, 2H, Ph), 7.42-7.26(m, 1H, 18H, Ph), 5.56(s, 1H, CHPh), 4.90(AB, 2H, CH2Ph, *J*a=9.4Hz, *J*b=10.2Hz, δv=37.3Hz), 4.84(d, 1H, H-1, *J*=4.0Hz), 4.77(dd, 2H, CH2Ph, *J*=8.0Hz, 12.0Hz), 4.60(d, 2H, CH2Ph, *J*=12.0Hz), 4.21(dd, 1H, H-4, *J*=5.0Hz, 10.5Hz), 4.12(t, 1H, H-3, *J*=9.5Hz), 3.92(dt, 1H, H-5, *J*=9.5Hz, 5.0Hz), 3.70(t, 1H, H-6a, *J*=10.5Hz), 3.63(t, 1H, H-6b, *J*=9.5Hz), 3.56(dd, 1H, H-2, *J*=4.0Hz, 12.5Hz). 13C NMR: (500MHz, CDCl3)-δ139.0(PhØ), 138.6(PhØ), 137.8(PhØ), 137.4(PhØ), 129.3(Ph), 128.9(Ph), 128.8(Ph), 128.7(Ph), 128.6(Ph), 128.4(Ph), 128.3(Ph), 128.2(Ph), 127.9(Ph), 126.5(Ph), 101.7(CHPh), 97.1(C-1), 82.7(C-2), 79.5(C-6), 79.1(C-3), 75.8(CH2Ph), 73.9(CH2Ph), 69.8(CH2Ph), 69.5(C-4), 63.1(C-5). HRMS ES+ (m/z) [M+] found 538.2365, calculated 538.2355.

**Allyl α-***D***-galactopyranoside.**

*α-D*-Galactose (0.10g, 0.55mmol) was partially dissolved in allyl alcohol (3ml, 44.0mmol). To this suspension *para*-toluenesulphonic acid (0.01g, 0.06mmol) was then added and the resulting suspension stirred for 16hours at 90°C. Diethyl ether (5ml) was added and the mixture was left to sit overnight. This was then evaporated off and the resulting syrup azeotroped with hexane to remove allyl alcohol yielding 0.11g (99%) of crude material a yellow syrup.

**Allyl 4,6-*O*-benzylidene-α-***D***-glalactopyranoside. (7) [16]**

Crude Allyl α-*D*-galactopyranoside (0.70g, 3.08mmol) and *para*-toluenesulphonic acid (0.06g, 0.29mmol) was dissolved in dry DMF (20ml). Benzaldehyde dimethyl acetal (1.4mls, 9.38mmol) was then added and the resulting solution stirred at 70°C for 30hours. The solution was then poured onto ice (5g) diethyl ether (5ml) and NaHCO3 (5ml) forming a precipitate which was filtered and the remaining product then extracted with ethyl acetate (4x5ml) dried (MgSO4) and concentrated in vacuo. The resulting oil was then triturated with ethanol and filtered yielding 0.49g (57%) of pure product a white crystalline solid.

1H NMR: (500MHz, CDCl3)-δ7.51-7.49(m, 2H, Ph), 7.38-7.36(m, 3H, Ph), 5.99-5.85(m, 1H, All-2-H), 5.56(s, 1H, CHPh), 5.31(d, 1H, All-3-Ha, *J*=17.2Hz), 5.23(d, 1H, All-3-Hb, *J*=10.4Hz), 5.09(d, 1H, H-1, *J*=2.8Hz), 4.33(d, 1H, H-6a, *J*=7.6Hz), 4.29-4.27(m, 3H, All-1-Ha, H-3, H-6b), 4.11-4.07(m, 2H, All-1-Hb, H-4), 3.94(dd, 1H, H-2, *J*=2.6Hz, *J*=7.1Hz), 3.49-3.47(m, 1H, H-5). 13C NMR: (500MHz, CDCl3)- δ137.04(PhØ), 133.23(All-2-C), 128.98(Ph, 1C), 128.01(Ph, 2C), 126.01(Ph, 2C), 117.76(All-3-C), 101.90(CHPh), 97.99(C-1), 75.60(C-3), 69.79(C-2), 69.73(C-4), 68.95(All-1-C), 68.61(C-6), 61.03(C-5).

MS: FAB+ (m/z) 308.12 calculated 308.13.

**Selective Alkylation.**

**General synthetic procedure.**

The Allyl 4, 6-O-benzylidene-α-*D*-glalactopyranoside(0.04g, 0.13mmol) was dissolved in dry solvent (0.8ml) and to this tetrabutylammonium iodide (0.01g, 0.02mmol) was added followed by the protecting group. Hexane washed sodium hydride 60% in mineral oils (0.02g, 0.56mmol) was suspended in dry solvent (0.8ml) and added to the sugar solution. The resulting solution was then left stirring at 70˚C for 16hours. The reaction mixture was then allowed to cool and to this a methanol/water (1:1) solution was added to destroy the remaining NaH. To this then brine (5ml) was added and an extraction carried out with DCM (4x5ml) the organic phases were then combined, washed with 10% KCl solution (2x5ml), dried and concentrated to give the crude product which was then columned (eluent hexane: ethyl acetate, 75:25) to give pure product in the yields shown below.

**Allyl 4,6-*O*- benzylidene-2,3-di-*O*-*para* methoxybenzyl-α-***D***-galactopyranoside. (11a)**

Pure yield 0.03g (60%)

1H NMR: (500MHz, CDCl3)-δ7.51(d, 2H, PMB-H, *J*=9.6Hz), 7.35-7.25(m, 7H, Ph, PMB-H), 6.86-6.83(m ,4H, PMB-H), 5.99-5.85(m, 1H, All-2-H), 5.46(s, 1H, CHPh), 5.29(d, 1H, All-3-Ha, *J*=18.2Hz), 5.18(d, 1H, All-3-Hb, *J*=12.5Hz), 4.90(d, 1H, H-1, *J*=4.5Hz), 4.79-4.76(m, 2H, CH2Ph), 4.74-4.57(m, 2H, CH2Ph), 4.14-4.13(m, 3H, All-1-Ha, H-6a, H-3), 4,05-3,97(m, 4H, All-1-Hb, H-6b, H-2, H-4), 3.80(s, 3H, OMe), 3.79(s, 3H, OMe) 3.61(d, 1H, H-5, *J*=6.3Hz). 13C NMR: (500MHz, CDCl3)- δ159.21(PMB-PhØ), 159.12(PMB-PhØ), 137.88(PhØ), 133.90(All-2-C), 130.98(PMB-PhØ), 130.82(PMB-PhØ), 129.61(Ph, 2C), 128.86(Ph, 2C), 128.10(Ph, 2C), 126.40(Ph, 2C), 118.01(All-3-C), 113.70(Ph, 2C), 113.69(Ph, 2C), 101.14(CHPh), 97.04(C-1), 75.68(C-2), 75.08(All-1-C), 74.97(C-6), 73.24(CH2Ph), 71.95(CH2Ph), 69.43(C-3), 68.45(C-4), 62.70(C-5), 55.27(2CH3).HRMS ES+ (m/z) [M+] found 548.2412, calculated 548.2410.

**Allyl 4,6-*O*-benzylidene-2-*O*-*tert*-butyldimethylsilyl –α-***D***-glalactopyranoside. (8b)**

Pure yield-0.02g (38%)

[α]D20 99.3 (c 0.004, CHCl3), 1H NMR: (500MHz, CDCl3)-δ7.40-7.38(m, 2H, Ph), 7.24-7.13(m, 3H, Ph), 5.94-5.82(m, 1H, All-2-H), 5.39(s, 1H, CHPh), 5.19(d, 1H, All-3-Ha, *J*=18.45Hz), 5.09(d, 1H, All-3-Hb, *J*=10.3Hz), 4.96(d, 1H, H-1, *J*=2.8Hz), 4.16(d, 1H, H-6a, *J*=12.4Hz), 4.13(dd, 1H, All-1-Ha, *J*=1.2Hz, *J*=11.0Hz), 3.97-3.86(m, 5H, All-1-Hb, H-6b, H-2, H-3, H-4), 3.58(s, 1H, H-5), 0.79(s, 9H, tBut), 0.04(s, 3H, CH3), 0.02(s, 3H, CH3). 13C NMR: (500MHz, CDCl3)- δ137.92(PhØ), 133.80(All-2-C), 128.70(Ph, 1C), 128.05(Ph, 2C), 126.04(Ph, 2C), 117.87(All-3-C), 100.70(CHPh), 98.33(C-1), 76.70(C-3), 71.03(C-2), 69.44(C-6), 68.90(All-2-C), 68.72(C-4), 63.22(C-5), 25.77(tBut), 0.00(2CH3). MS electrospray (m/z)- [M+Na]+=445.3, [2M+Na]+=867.3.

**Allyl 4,6-*O*-benzylidene-3-*O*-*tert-*butyldimethylsilyl –α-***D***-galactopyranoside. (9b)**

Pure yield-0.02g (35%)

[α]D20 51.3 (c 0.011, CHCl3), 1H NMR: (500MHz, CDCl3)-δ7.39-7.37(m, 2H, Ph), 7.25-7.21(m, 3H, Ph), 5.95-5.79(m ,1H, All-2-H), 5.43(s, 1H, CHPh), 5.21(d, 1H, All-3-Ha, *J*=17.2Hz), 5.08(d, 1H, All-3-Hb, *J*=11.8Hz), 4.80(d, 1H, H-1, *J*=2.7Hz), 4.17-4.12(m, 3H, All-1-Ha, H-6a, H-3), 3.95-3.88(m, 4H, All-1-Hb, H-6b, H-2, H-4), 3.63(d, 1H, H-5, *J*=2.7Hz), 0.79(s, 9H, tBut), 0.03(s, 3H, CH3), 0.02(s, 3H, CH3). 13C NMR: (500MHz, CDCl3)- δ138.07(PhØ), 134.44(All-2-C), 129.47(Ph, 1C), 128.60(Ph, 2C), 126.67(Ph, 2C), 118.06(All-3-C), 101.68(CHPh), 99.32(C-1), 76.62(C-3), 71.24(C-2), 69.88(C-6), 69.62(All-1-C), 69.24(C-4), 63.20(C-5), 26.19(tBut), 0.00(2CH3). MS electrospray (m/z)- [M+Na]+=445.3, [2M+Na]+=867.3.

**Allyl 4,6-*O*-benzylidene-2-*O*-*ter t-*butyldiphenylsilyl-α-***D***-galactopyranoside. (8c)**

Pure yield-0.01g (20%)

1H NMR: (500MHz, CDCl3)-δ7.74-7.71(m, 3H, Ph), 7.338-7.26(m, 12H, Ph), 6.00-5.84(m, 1H, All-2-H), 5.42(s, 1H, CHPh), 5.34(d, 1H, All-3-Ha, *J*=17.2Hz), 5.20(d, 1H, All-3-Hb, *J*=14.9Hz), 4.68(d, 1H, H-1, *J*=3.4Hz), 4.19-4.08(m, 5H, All-1-Ha, H-6a,b, H-3, H-2), 4.02(d, 1H, All-1-Hb, *J*=10.35Hz), 3.92(dd, 1H, H-4, *J*=4.05Hz, *J*=10.0Hz), 3.69(d, 1H, H-5, *J*=3.2Hz), 1.09(s, 3H, CH3). 13C NMR: (500MHz, CDCl3)- δ136.02(3PhØ), 134.01(All-2-C), 133.39(Ph, 1C), 129.74(Ph, 2C), 128.07(Ph, 3C), 127.67(Ph, 3C), 127.63(Ph, 3C), 126.26(Ph, 3C), 117.26(All-3-C), 100.91(CHPh), 99.02(C-1), 79.90(C-4), 76.15(C-6), 71.35(All-1-C), 69.33(C-3), 68.83(C-2), 62.67(C-5), 26.98(CH3).

**Allyl 4,6-*O*-benzylidene-3-*O*-*ter t-*butyldiphenylsilyl-α-***D***-galactopyranoside. (9c)**

Pure yield-0.01g (20%)

1H NMR: (500MHz, CDCl3)-δ7.77-7.75(m, 2H, Ph), 7.53(d, 1H, Ph, *J*=9.3Hz), 7.53-7.29(m, 12H, Ph), 5.91-5.79(m, 1H, All-2-H), 5.22(s, 1H, CHPh), 5.15(d, 1H, All-3-Ha, *J*=12.1Hz), 5.12(d, 1H, All-3-Hb, *J*=9.3Hz), 5.03(d, 1H, H-1, *J*=3.4Hz), 4.13-4.00(m, 5H, All-1-Ha, H-6a,b, H-3, H-2), 3.79(d, 1H, All-1-Hb, *J*=17.1Hz), 3.63(d, 1H, H-4, *J*=5.0Hz), 3.39(d, 1H, H-5, *J*=3.3Hz), 1.09(s, 3H, CH3). 13C NMR: (500MHz, CDCl3)- δ135.96(3PhØ), 133.63(All-2-C), 129.82(Ph, 2C), 129.74(Ph, 2C), 128.74(Ph, 2C), 128.09(Ph, 3C), 127.73(Ph, 2C), 127.56(Ph, 2C), 126.10(Ph, 2C), 117.14(All-2-C), 100.56(CHPh), 98.40(C-1), 81.20(C-4), 76.20(C-6), 71.71(All-1-C), 69.11(C-2), 68.31(C-3), 62.97(C-5), 26.89(CH3)

Reference List

1. Garneau S, Qiao L, Chen L, Walker S et al.: Bioorg. Med. Chem. 2004, **12**: 6473-6494.

2. Sugawara F, Nakayama H, Ogawa T: Carbohyd. Res. 1982, **108**: C5-C9.

3. Maillard P, Huel C, Momenteau M: Tet. Lett. 1992, **33**: 8081-8084.

4. Lassaletta JM, Carlsson K, Garegg PJ, Schmidt RR: J. Org. Chem. 1996, **61**: 6873-6880.

5. Zhang SQ, Li ZJ, Wang AB, Cai MS et al.: Carbohyd. Res. 1998, **308**(3-4): 281-285.

6. Khan SH, Abbas SA, Matta KL: Carbohyd. Res. 1989, **193**: 125-139.

7. Huang CY, Cabell LA, Anslyn EV: J. Am. Chem. Soc. 1994, **116**: 2778-2792.

8. Sanders WJ, Manning DD, Koeller KM, Kiessling LL: Tetrahedron 1997, **53**: 16391-16422.

9. Jacquinet JC, Petitou M, Duchaussoy P, Lederman I et al.: Carbohyd. Res. 1984, **130**: 221-241.

10. Yamada H, Harada T, Takahashi T: J. Am. Chem. Soc. 1994, **116**: 7919-7920.

11. Lefeber DJ, Kamerling JP, Vliegenthart JFG: Chem. Eur. J. 2001, **7**: 4411-4421.

12. Ishido Y, Sakairi N, Sekiya M, Nakazaki N: Carbohyd. Res. 1981, **97**: 51-79.

13. Cervi G, Peri F, Battistini C, Gennari C et al.: Bioorg. Med. Chem. 2006, **14**: 3349-3367.

14. Magnusson G, Ahlfors S, Dahmen J, Jansson K et al.: J. Org. Chem. 1990, **55**: 3932-3946.

15. Lubineau A, Thieffry A, Veyrieres A: Carbohyd. Res. 1976, **46**: 143-148.

16. Jacquinet JC, Sinay P: Tetrahedron 1979, **35**: 365-371.
